# Hybrid Recommendation Network Model with a Synthesis of Social Matrix Factorization and Link Probability Functions

**DOI:** 10.3390/s23052495

**Published:** 2023-02-23

**Authors:** Balraj Kumar, Neeraj Sharma, Bhisham Sharma, Norbert Herencsar, Gautam Srivastava

**Affiliations:** 1 School of Computer Application, Lovely Professional University, Phagwara 144411, Punjab, India; 2 Department of Computer Science, Punjabi University, Patiala 147002, Punjab, India; 3 Chitkara University Institute of Engineering and Technology, Chitkara University, Rajpura 140401, Punjab, India; 4 Department of Telecommunications, Faculty of Electrical and Communication Engineering, Brno University of Technology, Technicka 12, 616 00 Brno, Czech Republic; 5 Department of Mathematics and Computer Science, Brandon University, Brandon, MB R7A 6A9, Canada; 6 Department of Computer Science and Mathematics, Lebanese American University, Beirut 1102, Lebanon; 7 Research Centre for Interneural Computing, China Medical University, Taichung 40402, Taiwan

**Keywords:** collaborative filtering, topic modelling, recommendation system, collaborative topic regression, social matrix factorization, social network, item network structure

## Abstract

Recommender systems are becoming an integral part of routine life, as they are extensively used in daily decision-making processes such as online shopping for products or services, job references, matchmaking for marriage purposes, and many others. However, these recommender systems are lacking in producing quality recommendations owing to sparsity issues. Keeping this in mind, the present study introduces a hybrid recommendation model for recommending music artists to users which is hierarchical Bayesian in nature, known as Relational Collaborative Topic Regression with Social Matrix Factorization (RCTR–SMF). This model makes use of a lot of auxiliary domain knowledge and provides seamless integration of Social Matrix Factorization and Link Probability Functions into Collaborative Topic Regression-based recommender systems to attain better prediction accuracy. Here, the main emphasis is on examining the effectiveness of unified information related to social networking and an item-relational network structure in addition to item content and user-item interactions to make predictions for user ratings. RCTR–SMF addresses the sparsity problem by utilizing additional domain knowledge, and it can address the cold-start problem in the case that there is hardly any rating information available. Furthermore, this article exhibits the proposed model performance on a large real-world social media dataset. The proposed model provides a recall of 57% and demonstrates its superiority over other state-of-the-art recommendation algorithms.

## 1. Introduction

Recently, there has been huge information growth on the Internet due to the swift development of web applications and internet-based services. Internet users are struggling to access selective and relevant information due to data abundance [1]. Moreover, the availability of information on the Internet causes hindrances in decision-making processes. This is what is recognized as the information overload dilemma [2]. Generally, this situation arises when systems cannot manage big data systematically. In such cases, users may miss useful information and possibly access inappropriate and uninteresting content [3]. This is where Recommender Systems (RS) come into the picture as a helping tool to suggest various products and services to target users [4]. RS aim at generating recommendations by using machine learning (ML) algorithms for products or items as per users’ interest based on their records or preferences. RS are profoundly utilized in diverse areas such as advertisements, e-commerce, scientific articles, etc. RS are intelligent applications that have made significant contributions in numerous commercialized settings such as Netflix, Last.fm, Amazon, PrimeVideo, etc. [5].

In general, researchers classify ML-based recommenders as collaborative filtering [6,7], content-based [8], and hybrid [9,10]. The oldest and the most popular technique, Collaborative Filtering (CF) recommends items using records of similar users. CF techniques are categorized into two groups: neighbourhood-based (memory-based) [11,12] and model-based techniques [13,14]. Model-based techniques are also known as latent factor models. In the literature, model-based techniques are considered to have an edge over memory-based techniques owing to better performance and recommendation results. The major reason behind this is that model-based techniques fit a statistical model based on the training dataset, whereas memory-based techniques use the entire dataset and perform the weighted average of rating data to produce a recommendation. Therefore, owing to the superiority of latent factor models, these models are emphasized in this research. Content-based filtering employs item descriptions and features along with user profiles for the recommendation task [15]. Hybrid RS use ensemble approaches to integrate CF and content-based techniques to recommend items.

People are more active on social networking sites nowadays which include Facebook, LinkedIn, YouTube, Last.fm, Twitter, etc., where users can connect with their friends or other individuals having common interests while also being able to share different multimedia content such as viewpoints, small video clips, pictures, music, news, etc. Social networking sites have brought people closer together across the globe and can strongly influence the thinking pattern and decision-making of online users. Therefore, these social connections can be fruitful in improving the recommendation quality of RS such as recommendations for products, online music, news, or promotional content as claimed in [16,17]. Similarly, connections can also be explored among items that are to be recommended to users. These item relations can significantly contribute to recommendation tasks [18]. For instance, to recommend artists to users in Last.fm, the relations among artists are informative and can help recommend artists that create similar music. There are other examples also where relations among items can be determined. These include webpage hyperlinks, scientific articles written by the same authors, movies starring the same actors, etc. Thus, the prime focus of the present study is to demonstrate the effectiveness of unified information related to social networks and an item-relational network structure in addition to item content and user-item interactions to achieve better prediction accuracy.

The proposed algorithm uses different criteria to recommend artists to the target users as follows:

- The existing or old artists are noteworthy, as they are the ones who are well established and have made great contributions in their areas, and there are also the people who have a passionate love for old music. Thus, old artists are recommended based on other users’ preferences. Hence, traditional collaborative filtering methods are the best fit for this criterion.
- New or unheard artists are equally important as old ones. When a new album or new tracks are released, generally music lovers show a keen interest in the latest and new songs just to keep abreast of music in their interest. Therefore, because of new tracks or albums, there is no or very limited information available about user choices, and that makes it challenging for CF methods to make any recommendations. Thus, for recommending new or unheard artists, item content or attributes and item network structure information make major contributions in such cases.
- If a user is inactive or completely new to the system and a very small piece of information is known about their taste or there is no availability of their preferences, then it is possible to make effective use of relations among items as well as relations with other users on the social networks to make recommendations.
- Exploratory variables are significant for online user communities. Based on the content information of artists and the social relations of users, user profiles can be created to establish communities with similar preferences. Moreover, it is also possible to describe which artists are liked by which types of users.

### 1.1. Motivation

Recommendation technology generates a good sense of motivation to carry out the proposed research while keeping in view a specific dimension at the appropriate level of granularity alongside RS challenges. This research has been inspired by the work of Collaborative Topic Regression (CTR) [19], Collaborative Topic Regression with Social Matrix Factorization (CTR–SMF) [16], and Relational Collaborative Topic Regression (RCTR) models [20]. To alleviate the sparsity issue, CTR was proposed by integrating feedback information into item content information. CTR–SMF extended CTR further by integrating user network information. Likewise, the RCTR model extended CTR by integrating one more type of information, i.e., relational information of items into CTR. Following the same path, this study looks to merge the works of both CTR–SMF and RCTR. Therefore, the key purpose of this paper is to develop a hybrid hierarchical Bayesian recommendation model that aims to recommend artists to target users. Our methodology employs some auxiliary information including item network structure, user relations information, feedback, and item content information to enhance the accuracy of predictions. Therefore, this model is a joint graphical model that combines RCTR and CTR–SMF models. Such a model may fulfill the natural dual need of service providers and users with the automated generation of recommendations based on data analysis.

### 1.2. Problem Statement

In a recommendation problem, there are primarily two entities involved, i.e., users and items. In the present research, users are music lovers and items are the artists whose tracks are played by users. Suppose the recommendation system is expected to recommend artists to the users of their interest. Just like [19], assume *i* indicates users and *j* indicate items. Here $r_{ij}$ ∈ {0, 1} represents the case of whether user *i* has played artist *j*, where $r_{ij}$ is the rating variable. The track of the artist played indicates the preference of user *i* for artist *j* which means $r_{ij}$ = 1. However, if $r_{ij}$ = 0, then there are two different interpretations of this. First, user *i* has no preference for artist *j* and second, user *i* is unaware of artist *j*. That means it is not sure whether user *i* dislikes or is ignorant about artist *j*. The proposed model is based on the same settings (i.e., implicit ratings) as introduced in [21] and further used in [19]. The proposed recommendation model is flexible enough to be easily adjusted for explicit ratings given on a different scale as well. As discussed earlier, traditional collaborative filtering approaches rely upon a user preference rating matrix only and the rating matrix is expressed as {$r_{ij}$|*i* = 1, 2, …, *I*; *j* = 1, 2, …, *J*} [22]. However, in the rating matrix, ratings provided by users against a large number of items are very few which affects the performance of collaborative filtering techniques adversely. The lack of sufficient rating data generates the sparsity problem which in turn causes issues in producing quality recommendations. To address this issue, the proposed model takes into consideration a lot of auxiliary domain information. The main emphasis of this study is to examine the effectiveness of unified information related to social networking and item relational network structure in addition to item content and user-item interactions to make predictions for user ratings.

### 1.3. Contribution

This paper’s major contributions of this paper are as follows:

- The core contribution of this study is the further extension of RCTR and CTR–SMF models to build a hybrid hierarchical Bayesian RCTR–SMF model that impeccably assimilates rating data, item content, social information of users and relational information of items to mitigate the sparsity issue in RS.
- Another major contribution of RCTR–SMF is to demonstrate the effectiveness of item relational networks and social networks together in enhancing prediction accuracy.
- The RCTR–SMF model can address the cold-start problem in case there is hardly any rating information available. It makes effective use of item content or attributes, relations among items as well as relations with other users on the social networks to generate predictions for new users (who have rated very few items) and new items (with one or two ratings only). This, in turn, enhances the recommendation quality.
- The experiments conducted on a public dataset reveal that the proposed recommendation model can attain higher accuracy in predictions than state-of-the-art algorithms.

This paper is structured as follows: Section 2 emphasizes the review of the most relevant and essential related work. The proposed recommendation model is presented in Section 3. Section 4 highlights the experimental setup, demonstrates experiments on a public dataset and analyzes the findings. Section 5 provides a comparative analysis and confirms the strength of the proposed model over other recommendation methods. Finally, Section 6 wraps up the paper by presenting concluding remarks.

## 2. Related Works

This section presents the background of the proposed model (RCTR–SMF) and the related works in brief. This includes matrix factorization, topic modelling, CTR and other CTR-based approaches.

### 2.1. Matrix Factorization

CF predicts the interests of a target user using the preferences of other users. Matrix Factorization (MF) [23,24] and its extension Probabilistic Matrix Factorization (PMF) [23] are the most successful recommendation approaches of CF-based methods. MF and PMF are the leading approaches among latent factor models which are known for their auspicious performance. Matrix factorization identifies latent factors from the user-item interactions (ratings) matrix and performs the mapping of users and items against those latent factors. The prime notion of MF involves the usage of latent vectors to represent users and items in a low-dimensional space with dimension *K*. Thus, user *i* is characterized by a latent vector $u_{i}$ ∈ *R^K^* and item *j* by $v_{j}$ ∈ *R^K^*. The prediction for the item *j* likely to be given by user *i* can be calculated as in Equation (1):

(1) $${\hat{r}}_{ij}=u_{i}^{T}v_{j}$$

Let $U={\left(u_{i}\right)}_{i=1}^{I}$ and $V={\left(v_{j}\right)}_{j=1}^{J}$ be the latent matrices to represent hidden vectors for all users and items, respectively. In matrix factorization to minimize prediction error (loss function), we can optimize the objective function in Equation (2) to find the optimal U and V latent matrices [20]:

(2) $$min_{U,V}=\sum_{i=1}^{I}\sum_{j=1}^{J}{\left(r_{ij}-u_{i}^{T}v_{j}\right)}^{2}+\lambda_{u}\sum_{i=1}^{I}∥u_{i}{∥}^{2}+\lambda_{v}\sum_{j=1}^{J}∥v_{j}{∥}^{2}$$

where $\lambda_{u}$ and $\lambda_{v}$ are the regularized tuning parameters to manage the complexity of the model and can range from 0 to ∞. Here, 0 means no effect and ∞ means maximum effect. Regularization is a technique to avoid overfitting problems by reducing the regularized squared error. Therefore, the magnitude of coefficients (learned parameters) is penalized because regularization forces them toward 0.

The maximum a posteriori (MAP) estimates the PMF model [23] corresponds to the objective function in Equation (2). The authors in [19] generalized the PMF model as in Equation (3):

(3) $$u_{i}\;\sim \;N\left(0,\lambda_{u}^{-1}I_{K}\right),v_{j}\;\sim \;N\left(0,\lambda_{v}^{-1}I_{K}\right),r_{ij}\;\sim \;N\left(u_{i}^{T}v_{j},c_{ij}^{-1}\right)$$

where $I_{K}$ is the identity matrix with *K* dimensions and $c_{ij}$ is the precision or confidence parameter for $r_{ij}$ and is defined as in Equation (4):

(4) $$c_{ij}=\left\{\begin{array}{c} \;a,\;\;\;\;if\;r_{ij}=1,\\ \;b,\;\;\;\;if\;r_{ij}=0, \end{array}\right.$$

where *a* and *b* are tuning parameters when *a* > *b* > 0. If $c_{ij}$ = 1, MAP estimate matches with a solution of Equation (2). The larger the value of $c_{ij}$, the more trust is in $r_{ij}$.

Matrix factorization techniques are known for their strong performance, however, are not free from sparsity problems and find it difficult to address out-of-matrix prediction. Besides this, in matrix factorization, the interpretation of learnt latent space is also difficult [19,20].

### 2.2. Topic Modeling

Topic modelling in machine learning [25] can be described as an unsupervised statistical modelling technique that can be utilized to detect a set of latent “topics” from an enormous document collection. The “topic” here is distributed across terms inclined towards a particular theme or a subject. Such a discovery of topics is purely performed considering a hierarchical Bayesian analysis of a given text. Its main use is in text-mining where it is used to find out the hidden semantic patterns in text. The hidden topic model LDA, i.e., Latent Dirichlet Allocation [26], the simplest topic model, helps to discover topics automatically. LDA assumes that topics are produced before documents [27]. Probabilistic topic modelling, an extended form of topic modelling, is characterized by a collection of algorithms whose objective is to discover and annotate enormous sets of documents based on diverse themes. These themes may include education, games, culture, international affairs, domestic industries, politics, etc. The application areas where these modelling tools have major contributions are information retrieval, document classification and corpus exploration.

The basic objective of using topic modelling in RS are to provide content-based modelling of items. When the corpus of documents is ready, the variational Expectation–Maximization (EM) method can be applied to learn topics and then, documents can be decomposed accordingly [26]. For any new document, a variational EM algorithm can be used to infer topics from the contents of a given item.

### 2.3. CTR and Its Variants

CTR is the first hybrid recommendation approach of its kind that uses a user rating matrix along with item content for recommending research articles to other researchers/authors. CTR’s basic purpose is to fit a model by integrating the MF-based CF technique with probabilistic topic modelling and to employ the latent topic space to describe noted words and noted ratings [19]. CTR extracts the users’/items’ latent features from user rating data and uses item content information to record the distribution of topics of items [28]. Thus, the topic proportions $\theta_{j}$ can be replaced with a latent item vector $v_{j}$ in Equation (3) to obtain Equation (5):

(5) $$r_{ij}\;\sim \;N\left(u_{i}^{T}\theta_{j},c_{ij}^{-1}\right)$$

**Figure 1:**
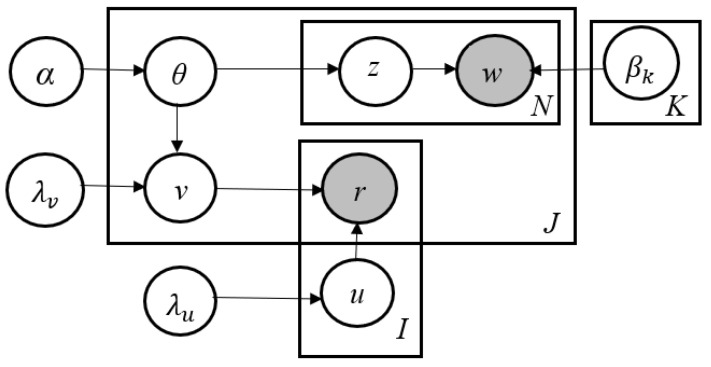
CTR Model.

CTR also addresses the problems of MF-based collaborative filtering techniques. With this, CTR can outperform MF-based CF approaches with improved interpretability of results essential for recommendations. Figure 1 presents the graphical model of CTR [19]. This model uses one extra latent variable (i.e., item latent offset) $\epsilon_{j}$ between item latent vectors $v_{j}$ in collaborative filtering and topic proportions $\theta_{j}$ in latent Dirichlet allocation. This offset represents the gap between what a research paper is actually about and what researchers understand about it. The offset can be understood better when there are adequate user ratings available.

The item latent offset $\epsilon_{j}$ plays a key role in CTR. It attempts to make an item a latent vector $v_{j}$ closer to topic proportions $\theta_{j}$ and then possibly deviate from it if required. $\lambda_{v}$ is the regularization parameter to monitor how close $v_{j}$ is to $\theta_{j}$.

CTR Variants: CTR is a hybrid recommendation approach that uses the user rating matrix along with item content for recommending research articles to other researchers/authors [19]. However, it suffers from a cold-start problem in the absence of user ratings (high sparsity of ratings). To tackle sparsity and cold-start issues, several researchers have extended the work of CTR and have come up with enhanced models. There are two models in particular, CTR–SMF and CTR–SMF2, proposed in [16,17], respectively, that integrate social information into CTR to highlight the contribution of social relations in boosting recommendation quality. LA–CTR, another extended variant, works on the principle that users’ limited attention gets divided non-uniformly among people [29]. CSTR uses social network information extensively for recommending celebrities to general users [30].

The authors in [20] proposed RCTR that extends CTR by fusing item network structure information into CTR to improve recommendation accuracy. The authors in [31] presented a CTR-based time-aware recommendation model T-CTR to recommend scientific articles. SICTR uses users’ latent features based on their social relationships and topics which show their active participation [32]. The TagCDCTR model employs tag sharing to connect related domains with a collaborative-topic-modelling approach [33]. The authors in [28] proposed a novel CTR-based three-way recommender model and designed a PMF-LDA-CTR-based granulation strategy to mine granular features and recognize interpretable multi-level recommendations. Although the above-mentioned CTR-based methods have demonstrated improvements in different aspects, there is still an open issue of an effective fusion of social network information with item network structure into CTR that is being emphasized here in the current study. A system for detecting and classifying a 3000 image dataset of LCC disease based on four different disease levels has been developed using deep learning (DL) based convolutional long-term network (CLTN) amalgamated model of convolutional neural networks (CNN) and long short-term memory (LSTM). Lemon citrus canker (LCC) is one of those diseases that has a draconian effect on lemon production [34]. Through the southbound application programming interface, all information is provided to data paths or data elements like network switches and routers, and through the northbound application programming interface, information is provided to applications like firewalls, load balancers, and business logic. The SDN controller provides flexibility to create numerous new applications since it is positioned in the middle of the architecture between the network components and SDN applications [35].

## 3. Proposed Model

This section presents the proposed recommendation model, called Relational Collaborative Topic Regression with Social Matrix Factorization (RCTR–SMF). The RCTR–SMF model is hybrid hierarchical Bayesian in nature and creates a fusion of RCTR and CTR–SMF models. The primary goal of this model is to use different types of auxiliary information, as utilized by RCTR and CTR–SMF, to boost the accuracy of predictions and in turn enhance the quality of recommendations. This section provides the essential details needed to build the proposed model followed by parameter learning using a Maximum A Posteriori estimate. Then, the computational procedure used is described for making predictions. At last, the model provides an overview of a family of Link Probability functions.

### 3.1. Model Building

**Figure 2:**
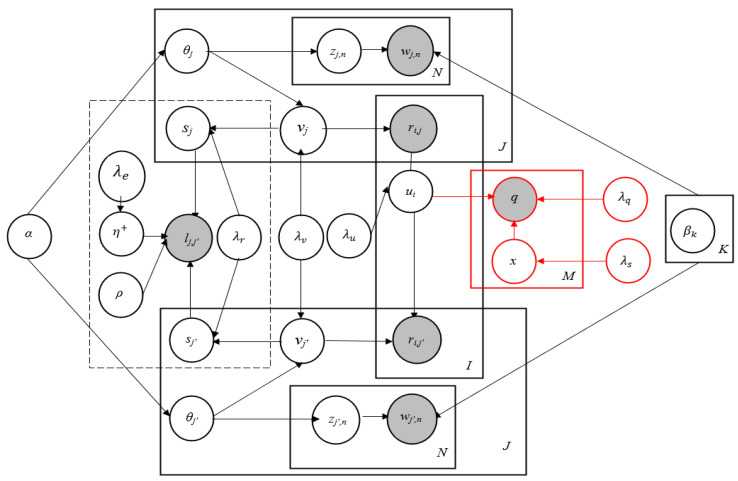
RCTR–SMF Model.

To demonstrate the graphical model of RCTR–SMF, the same technique is followed here as adopted in [20]. The graphical model of the proposed model, i.e., RCTR–SMF, is presented in Figure 2. In this figure, the RCTR part is demonstrated in black and SMF is represented in red.

The generative procedure of the proposed model is given as follows:

1. For each user *i*: draw a user latent vector $u_{i}\;\sim \;N\left(0,\lambda_{u}^{-1}I_{K}\right),$
2. For each item *j*:
  (a) Draw topic proportions $\theta_{j}$ ~ Dirichlet (*α*).
  (b) Draw item latent offset $\epsilon_{j}\;\sim \;N\left(0,\lambda_{v}^{-1}I_{K}\right)$ and set the item latent vector as
    $$v_{j}=\epsilon_{j}+\theta_{j}.$$
  (c) Draw item relational offset $\tau_{i}\;\sim \;N\left(0,\lambda_{r}^{-1}I_{K}\right)$ and set the item relational vector as
    $$s_{j}=\tau_{j}+v_{j}.$$
  (d) For each word $w_{jn}$:
    (i) Draw topic assignment $z_{jn}$ ~ *Mult* (*θ_j_*).
    (ii) Draw word $w_{jn}\;\sim$ *Mult* ($\beta_{z_{jn}}$*).*
3. Draw parameter $\eta^{+}\;\sim \;N\left(0,\lambda_{e}^{-1}I_{K+1}\right),$
4. Draw a binary link pointer between each pair of items ($j,\;j^{\prime }$),
  $$l_{j,\;j^{\prime }}|\;s_{j},\;s_{j^{\prime }}\sim \psi (\cdot |\;s_{j},\;s_{j^{\prime }},\;\eta^{+})$$
5. Draw the rating for each user-item pair (*i*, *j*) as
  $$r_{ij}\;\sim \;N\left(u_{i}^{T}v_{j},c_{ij}^{-1}\right)$$

The Link Probability Function (LPF), in the above procedure, is defined in Equation (6):

(6) $$\psi \;(l_{j,\;j^{\prime }}=1|\;s_{j},\;s_{j^{\prime }},\eta^{+})={\left[\sigma \left(\eta^{T}\left(s_{j}\circ s_{j^{\prime }}\right)+v\right)\right]}^{\rho }$$

where $l_{j,\;j^{\prime }}$ can assume a binary value to represent item relations, $l_{j,\;j^{\prime }}$ = 1 indicates that a relation or a link exists between a pair of items (*j*, $j^{\prime }$*),* whereas $l_{j,\;j^{\prime }}$ = 0 indicates the absence of any relation, v (a scalar value) represents the offset, $\eta^{+}$ = 〈η,v〉 denotes the vector-scalar concatenation, ∘ is an operator that represents (element-wise) vector multiplication, and $\sigma \left(\cdot \right)$ defines the sigmoid function given in Equation (7):

(7) $$\sigma \left(x\right)=\frac{1}{1+e^{-x}}$$

In the above generative procedure, the item relational offset ($\tau_{j}$), the key property of RCTR, is like item latent offset ($\epsilon_{j}$), a key property of CTR. As per requirement, $\tau_{j}$ can cause $s_{j}$ for divergence from item latent vector $v_{j}$. Here, $v_{j}$ reflects the users’ thinking of what item *j* is about whereas the item relational vector $s_{j}$ reflects the impact of other items on item *j*. A higher value of $\lambda_{r}$ indicates that $v_{j}$ and $s_{j}$ are closer to each other. The model degenerates with $v_{j}$ = $s_{j}$ when $\lambda_{r}$ reaches to ∞. Experiments also confirm that the performance of the RCTR–SMF model is better than the degenerated model and validates the efficacy of the item relational offset $\tau_{j}$. One important point that needs to be noted is that to keep things simple and fair, the same Gaussian model has been adopted here as was used in [19,20].

### 3.2. Social Network Graph

Let *G* = (*V*, *E*) be a social network graph where the set of nodes *V* = ${\left\{v_{i}\right\}}_{i=1}^{m}$ and the set of edges *E* of *G* represent users and their social relationships, respectively. To represent the social network matrix here, let *Q =* $q_{ik}$ be the *m* × *m* matrix of *G*. Suppose $q_{ik}$ for any pair of nodes ($v_{i}$, $v_{k}$) represents the relationship between two users (*i*, *k*). Then, $q_{ik}$ is connected with a confidence parameter $d_{ik}$ to represent the relation strength of users. A large value of $d_{ik}$ represents a stronger relationship between two users (*i*, *k*). Thus, the key goal of SMF is basically to examine the social network graph G to create a users’ l-dimensional feature space.

Let *U* ∈ *R^l × m^* be the user latent matrix and *S* ∈ *R^l × m^* be the social factor feature matrix. Additionally, let *U_i_* be the user-specific latent vector and *S_k_* be the social factor-specific latent feature vector. Equation (8) provides the conditional probability distribution over the observed social relations:

(8) $$P\left(Q|U,S,\sigma_{Q}^{2}\right)=\prod_{i=1}^{m}\prod_{k=1}^{m}N{\left(q_{ij}|\sigma \left(U_{i}^{T}S_{k}\right),\sigma_{Q}^{2}\right)}^{I_{ij}^{Q}}$$

where N(x|$\;\mu ,\;\sigma^{2}$) indicates a normal distribution, μ is the mean and $\sigma_{Q}^{2}$ is variance. $I_{ik}^{Q}$ is a function of social relations with binary values. If $I_{ik}^{Q}$ is 1, this indicates an edge (or a link) between the pair of nodes (*i*, *k*), i.e., user *i* is connected with user *k* in the social graph, and if $I_{ik}^{Q}$ is 0, then no edge (or a connection/link) exists between the pair of nodes (*i*, *k*). $\sigma \left(\cdot \right)$ is the sigmoid function as given above that limits the range of $U_{i}^{T}S_{k}$ within [0,1]. 0-mean spherical Gaussian priors are then placed on user and social factor feature vectors, as shown in Equations (9) and (10):

(9) $$P(U\;|\;\sigma_{U}^{2})\;=\;\prod_{i=1}^{m}\;N(U_{i}\;|0,\sigma_{U}^{2}I)$$

(10) $$P(S\;|\;\sigma_{S}^{2})\;=\;\prod_{k=1}^{m}\;N(S_{k}\;|0,\sigma_{S}^{2}I)$$

Thus, with Bayesian inference in Equation (11):

(11) $$p(U,\;S|Q,\sigma_{Q}^{2},\;\sigma_{U}^{2},\;\sigma_{S}^{2})\;\propto \;p(Q|U,\;S,\;\sigma_{Q}^{2})p(U|\sigma_{U}^{2})p(S|\sigma_{S}^{2})$$

When LDA is combined with SMF, as shown in Equation (12):

(12) $$p(U\;,V\;,S|Q\;,\;R,\;\sigma_{Q}^{2},\;\sigma_{R}^{2},\sigma_{U}^{2},\;\sigma_{V}^{2},\sigma_{S}^{2})\;\propto \;p(R|U,V,\;\sigma_{R}^{2})p(Q|U,S,\;\sigma_{Q}^{2})\;\times \;p(U|\sigma_{U}^{2})p(V|\sigma_{V}^{2})p(S|\sigma_{S}^{2})$$

To find the log of the posterior distribution of Equation (12), the substitution of the corresponding pdfs is required. To generate the item latent vector $v_{j}$, a key property as adopted in CTR is also used here in Equation (13):

(13) $$P(V|\sigma_{V}^{2})\;\sim \;N\left(\theta_{j},\lambda_{V}^{-1}I_{K}\right)$$

where $\lambda_{V}=\sigma_{R}^{2}/\sigma_{V}^{2}$.

### 3.3. Learning the Parameters

In the proposed model, there is the possibility that all parameters may be considered as random variables, and hence a fully Bayesian technique can be adopted for learning and inference [36]. However, it is not done here due to the very high computational cost. Since the fundamental objective of this research is to demonstrate the fusion of various kinds of auxiliary information to boost recommendation accuracy, it is obvious to follow the same learning strategy for learning and inference as used in CTR, RCTR and CTR–SMF models. In addition to this, a Maximum A Posteriori (MAP) estimate is adopted in base models and is also followed here for parameter learning. MAP attempts to maximize the log-posteriori of *U, V*, $\eta^{+}$, *s*1:*J*, *θ*1:*J*, and β, when the hyper-parameters ρ, $\lambda_{u}$, $\lambda_{v}$, $\lambda_{r}$, and $\lambda_{e}$ are given as specified in Equation (14):

(14) $$\begin{array}{cc} L= & \rho \sum_{l_{j,j^{\prime }}}\log\sigma \left(\eta^{T}\left(s_{j}\circ s_{j^{\prime }}\right)+v\right)-\frac{\lambda_{u}}{2}\sum_{i}u_{i}^{T}u_{i}\\ \; & -\frac{\lambda_{v}}{2}\sum_{j}{\left(v_{j}-\theta_{j}\right)}^{T}\left(v_{j}-\theta_{j}\right)\\ \; & -\frac{\lambda_{r}}{2}\sum_{j}{\left(s_{j}-v_{j}\right)}^{T}\left(s_{j}-v_{j}\right)-\frac{\lambda_{e}}{2}\eta^{+T}\eta^{+}\\ \; & +\sum_{j}\sum_{n}\log\left(\sum_{k}\theta_{jk}\beta_{k,w_{jn}}\right)\\ \; & -\sum_{i,j}\frac{c_{ij}}{2}{\left(r_{ij}-u_{i}^{T}v_{j}\right)}^{2} \end{array}$$

Like CTR and RCTR, a constant is omitted and the Dirichlet prior (α, i.e., the hyperparameter) of the topic model is set to 1. Then, the coordinate ascent is used to optimize this objective function. Here, an alternate algorithm is developed to learn parameters because L does not take the convex shape when all the variables are put together. That is why only one parameter gets optimized at a time, keeping all other parameters fixed.

Now, set the gradient to 0 to obtain the updated rules for $u_{i}$ and $v_{j}$, as given in Equations (15) and (16):

(15) $$u_{i}={\left(VC_{i}V^{T}+\lambda_{Q}SD_{i}S^{T}+\lambda_{u}I_{k}\right)}^{-1}+\left(VC_{i}R_{i}+\lambda_{Q}SD_{i}Q_{i}\right)$$

(16) $$v_{j}\leftarrow {\left(UC_{i}U^{T}+\lambda_{v}I_{K}+\lambda_{r}I_{K}\right)}^{-1}\left(UC_{j}R_{j}+\lambda_{v}\theta_{j}+\lambda_{r}s_{j}\right)$$

where $C_{i}$ and $D_{i}$ are the diagonal matrices with {$c_{ij}$| j = 1, 2, …, *J*} ($c_{ij}$ signifies the confidence managed by tuning parameters *a* and *b*, as given in [21] and {$d_{ij}$| j = 1, 2, …, *J*}, respectively, and *Ri* = {$r_{ij}$| j = 1, 2, …, *J*} being a column-vector having the ratings by user *i*.

In the context of $s_{j}$ and $\eta^{+}$, first, the variables are updated using gradient ascent, then the gradients of L w.r.t. $\;s_{j}$ or $\eta^{+}$ are taken. Taking the gradient of L w.r.t. $\;s_{j}$ as in Equation (17):

(17) $$\nabla_{\;s_{j}}\;L\;=p\sum_{l_{j,\;j^{\prime }}=1}(1-\sigma \left(\eta^{T}\left(s_{j}\circ s_{j^{\prime }}\right)+v\right)\left(\eta \;ο\;s_{j^{\prime }}\right)\;-\;\lambda_{r}(s_{j}-v_{j}).\;$$

Now, taking the gradient of L w. r. t. $\eta^{+}$ as in Equation (18):

(18) $$\nabla_{\eta^{+}\;}L\;=p\sum_{l_{j,\;j^{\prime }}=1}\left(1-\sigma \left(\eta^{+}{}^{T}\pi_{j,\;j^{\prime }}^{+}\right)\right)\pi_{j,\;j^{\prime }}^{+}-\lambda_{e}\eta^{+},$$

where $\pi_{j,\;j^{\prime }}^{+}$ = 〈$s_{j}\;\circ \;s_{j^{\prime }},1$〉.

For $\theta_{j}$, define q($z_{jn}$ = k) = $\psi_{jnk}$, get the items separated that contain $\theta_{j}$ and then use Jensen’s inequality method, as in Equation (19):

(19) $$L(\theta_{j})\;\geq -\frac{\lambda_{v}}{2}\;{\left(v_{j}-\theta_{j}\right)}^{T}\;(v_{j}-\theta_{j})+\;\sum_{n}\sum_{k}\phi_{jnk}(\log\;\theta_{jk}\;\beta_{k,w_{jn}}-\;\log\;\phi_{jnk})\;=\;L(\theta_{j},\phi_{j}).$$

Consider $\phi_{j}$ = ${\left(\phi_{jnk}\right)}_{n=1,k=1}^{N\times K}$. It is evident that L($\theta_{j},\phi_{j}$) gives a tight lower bound of L($\theta_{j}$) and $\theta_{j}$ can be optimized using projection gradient. The optimal $\phi_{jnk}$ is given in Equation (20):

(20) $$\phi_{jnk}\propto \theta_{jk}\;\beta_{k,w_{jn}}$$

To optimize β, apply the M-step update in Equation (21), exactly as used in LDA [26]:

(21) $$\beta_{kw}\propto \sum_{j}\sum_{n}\phi_{jnk}\;1\left[w_{jn}=w\right].$$

### 3.4. Prediction

Once all optimal parameters are successfully learnt, in-matrix and out-of-matrix predictions for the proposed model (RCTR–SMF) can be made. Suppose *D* is the observed testing dataset; to determine in-matrix predictions exactly as in [19], the point estimate of $u_{i}$, $\theta_{j}$ and $\epsilon_{j}$ has been used here to compute the predicted ratings as given in Equations (22)–(24):

(22) $$E[r_{ij}|D]\;\approx \;\;E{\left[u_{i}|D\right]}^{T}\left(E[\theta_{j}|D\right]+E[\epsilon_{j}|D]),$$

(23) $${\hat{r}}_{ij}=u_{i}^{T}v_{j}\;$$

(24) $$r_{ij}^{*}={\left(u_{i}^{*}\right)}^{T}(\theta_{j}^{*}+\epsilon_{j}^{*})={\left(u_{i}^{*}\right)}^{T}v_{j}^{*}\;$$

where *E(*·*)* represents the expectation function.

To determine the out-of-matrix predictions for unseen items which have no ratings, the ratings are predicted using Equation (25):

(25) $$∵\;E[\epsilon_{j}]=0,\;r_{ij}^{*}={\left(u_{i}^{*}\right)}^{T}\theta_{j}^{*}$$

### 3.5. About the Link Probability Functions

**Figure 3:**
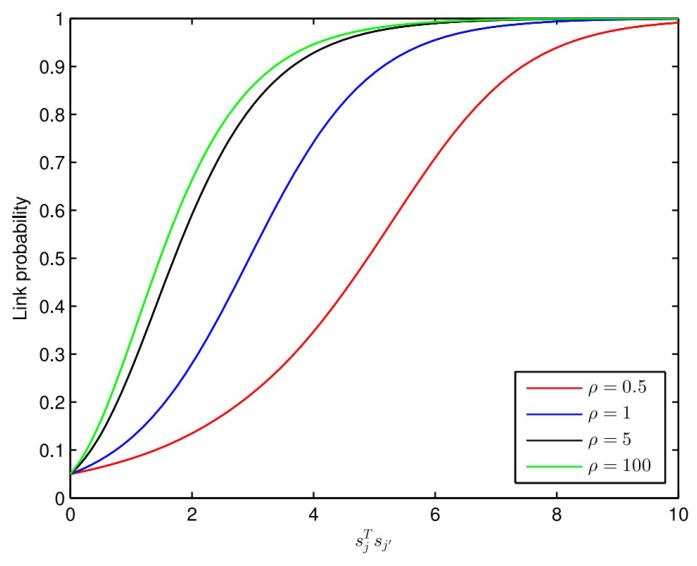
Plotting of link probability functions by varying ρ.

The Link Probability Functions (LPF) family influences the Relational Topic Model, i.e., RTM [36]. The prediction accuracy may vary depending upon the use of these functions. In the RCTR–SMF model, the selection of these LPF functions depends only on a single parameter, i.e., ρ. ρ being a non-negative real number, this LPF family holds an infinite number of such candidate functions. The authors in [37] proposed only two such functions, which is why new LPF functions may enhance the modelling capacity of the proposed model. Thus, ρ can be treated as a regularization hyperparameter from an optimization point of view. By varying the value of ρ, different LPF functions can be compared and flexibility in their behaviour can be observed as shown in Figure 3 [20]. Figure 3 plots probability curves using ψ ($l_{j,\;j^{\prime }}=1$| $s_{j}$, $s_{j^{\prime }},\eta^{+}$) = ${\left[\sigma \left(\eta^{T}\left(s_{j}\circ s_{j^{\prime }}\right)+v\right)\right]}^{\rho }$, when η = 1 and v is adjusted to ensure the same starting point for all link probability functions.

It can be observed from Figure 3 that when ρ = 1, the LPF used in RCTR–SMF collapses to one of the LPFs [37]. Besides this, CTR–SMF also uses Equation (26):

(26) $$\psi \;(l_{j,\;j^{\prime }}=1|\;s_{j},\;s_{j^{\prime }},\eta^{+})=\sigma (s_{j}{}^{T}s_{j^{\prime }})$$

if ρ = 1, v = 0 and η = 1 [16].

However, the LPF of RCTR–SMF becomes more flexible with v and η parameters as compared to CTR–SMF. Here the experiments confirm that all η elements are not the same and v ≠ 0. This implies that v and η are the essential parameters for deciding whether two items are connected or not.

## 4. Results Analysis and Discussion

This section of the paper highlights the experimental setup, key findings and results obtained through an experimental analysis conducted on the proposed recommendation model using a real-world dataset, Last.fm. In addition to this, it also deliberates on the complexity analysis of the proposed recommendation algorithm.

### 4.1. Experimental Analysis

This sub-section emphasizes the experimental setup used for conducting various experiments on the proposed recommendation algorithm. This includes a real-world dataset, experimental settings, and evaluation metrics used to perform analysis. First, a detailed description as well as necessary interpretations are provided of the dataset used. Then, experimental settings are highlighted where different values were set for different parameters to acquire a strong performance of the proposed system. Lastly, evaluation metrics used are examined where recall was preferred over precision due to ambiguity in the interpretation of zero ratings.

#### 4.1.1. Dataset Used

Experiments were performed on a large real-world social media dataset hetrec2011-lastfm-2k (Last.fm) [38]. The Last.fm (an online music service) dataset provides music artist listening information, social networking and tagging information from around 2K users. The description of this dataset is given in Table 1.

In the hetrec2011-lastfm-2k dataset, artists are considered as items. The sparsity level of the dataset is very high, i.e., 99.72%. In other words, the ratio of non-zero entries (i.e., the density of the dataset = 1—sparsity) is 0.0028. Initially, in the pre-processing stage, the dataset is cleaned to improve its quality by removing any unwanted noisy entries. In the Last.fm dataset, if a user listens to an artist (i.e., item), then the corresponding user rating for that artist is considered as 1. Otherwise, the user rating for that artist is considered missing and is indicated as 0.

#### 4.1.2. Experimental Settings

While performing experiments, a validation set was employed to obtain optimal parameters for matrix-factorization-based CF [31], CTR [19], CTR–SMF [16], and RCTR [20], respectively and strong performance was achieved using a grid search on the testing dataset. It was observed that all CF, CTR, CTR–SMF, and RCTR give good performance if $\lambda_{u}$ = 0.01, $\lambda_{v}$ = 100, *a* = 1, *b* = 0.01. Like CTR, CTR–SMF, and RCTR, here also *K* = 200 was considered. Here, *a* and *b* are taken as the tuning parameters *s.t.* a > b > 0 and are used to control the confidence parameters $c_{ij}$ and $d_{ij}$. For the proposed recommendation model, i.e., RCTR–SMF, the parameters $\lambda_{u}$ = 0.01, $\lambda_{v}$ = 100, *a* = 1, *b* = 0.01 and *K* = 200 were set and all other parameters were varied to understand their influence on the accuracy of predictions.

As in our proposed model and CTR, CTR–SMF, and RCTR as well, *M* which represents the number of recommended items can vary where *M* = {50, 100, 150, 200, 250, 300} in *recall@M* and *item_recall@M*. Small or large *M* may vary from application to application. In some cases, a smaller *M* may be more justified while in other cases a bigger *M* may be more meaningful. For example, a person may listen to the music of at least 50 artists in a span of 5 to 6 years. Moreover, the recall was observed to be too small across all the models on smaller values of M, especially when *M* < 50. The main reason is that in the testing data, the average count of artists per user is very low. That is why here in the present research *M* does not assume a value less than 50.

#### 4.1.3. Evaluation Metrics

An evaluation scheme was designed to evaluate the recommendation model in both cases, i.e., user-oriented and item-oriented scenarios. Two evaluation metrics can be used, i.e., precision and recall. However, in the context of the present research, only recall was identified as the appropriate performance evaluation measure because the precision metric is hard to evaluate accurately. The major reason is that a zero rating for an item is ambiguous and can indicate that the user has no preference for the item or is not aware of the existence of the item. Moreover, the case ratings $r_{ij}=1$ are taken up as true positives and recall uses only the positively rated items among the top *M*. Hence, the recall is in focus here. Therefore, all evaluations are performed by calculating the recall score that is mainly used for assessing accuracy. The *recall@M* for each user is described as given in Equation (27):

(27) $$recall@M=\frac{Number\;of\;items\;the\;user\;likes\;in\;top\;M}{Total\;number\;of\;items\;the\;user\;likes}$$

As usual, the predicted ratings of those items are sorted which are not the part of training data and the top *M* artists are recommended to the user. A recommender system is considered to be better if it achieves a higher recall value with a lower *M*. The mean score of recall putting all users together can be used to summarize the recall for the entire system. The evaluation strategy discussed so far applies to user-oriented scenarios.

To test the prediction capability of the recommendation system on a specific item, item-oriented recall is also computed. For item-oriented scenarios, a similar evaluation strategy can be used. The *item_recall@M* for each item is described as given in Equation (28):

(28) $$item\_recall@M=\frac{Number\;of\;users\;liked\;the\;item\;in\;top\;M}{Total\;number\;of\;users\;liked\;the\;item}$$

The purpose of this evaluation scheme is to measure the prediction performance of the system on a selected set of items.

### 4.2. Generating Top M Recommendations

The basic objective of a recommender system is to provide suggestions to the target user. This subsection provides a sample output of the proposed recommendation algorithm. Table 2 presents a list of the top 10 recommendations in decreasing order of prediction values generated by the proposed algorithm for a user with *userID* 45.

### 4.3. Complexity Analysis

The complexity analysis focuses on the running time of the proposed recommendation algorithm. As per the RCTR–SMF learning process (based on update rules), during each iteration, η updates require *O(KL)* time complexity, where *K* represents the latent-factor-space dimensionality and *L* represents the number of connections or relationships in the item relational network and social network. Likewise, the same cost *O(KL)* is also required to update the social matrix *Q =* ${\left(q_{i}\right)}_{i=1}^{I}$ and item relational matrix *S =* ${\left(s_{j}\right)}_{j=1}^{J}$ for each iteration. Other variable updates require the same cost as needed in [19]. The complexity required to update *U* is *O(IK3 + IJK2)* and to update *V* is *O(JK3 + IJK2)*, where *I* and *J* indicate the number of users and items, respectively. In RCTR–SMF, there is only the addition of *O(KL)* extra time compared to CTR during each iteration.

Based on the experiments, it can be concluded that satisfactory accuracy can be achieved by RCTR–SMF with a lesser number of learning iterations as compared to CTR but at par with CTR–SMF and RCTR. Hence, in comparison to CTR, even though the time complexity of each iteration of the proposed model is a little higher, overall (all iterations put together) the running time of training RCTR–SMF is lower.

## 5. Comparative Analysis and Discussions

Several experiments were designed and conducted, and performance comparisons of the proposed model RCTR–SMF were made with other recommendation algorithms. RCTR–SMF was evaluated on a large real-world social media dataset, Last.fm, to recommend music artists. Here the key questions to be answered based on the experiments include:

(a) How does the proposed model (i.e., RCTR–SMF) perform in comparison to other existing recommendation approaches?
(b) How does social matrix factorization and the family of LPFs together help improve prediction accuracy?
(c) How do the various parameters affect the prediction performance such as $\lambda_{v}$ (item content parameter), $\lambda_{r}$ (item relational parameter) and $\lambda_{q}$ (social relationship parameter)?

### 5.1. Performance Comparison

The performance of RCTR–SMF is evaluated and compared with other recommendation algorithms under a user-oriented scenario. Here, the main emphasis is on user-oriented over item-oriented recall due to simplicity, consistency, and convenience throughout the current study. For the performance comparison of RCTR–SMF, it was compared with other recommendation methods such as CF [7], CTR [19], CTR–SMF [16], and RCTR [20], respectively.

**Figure 4:**
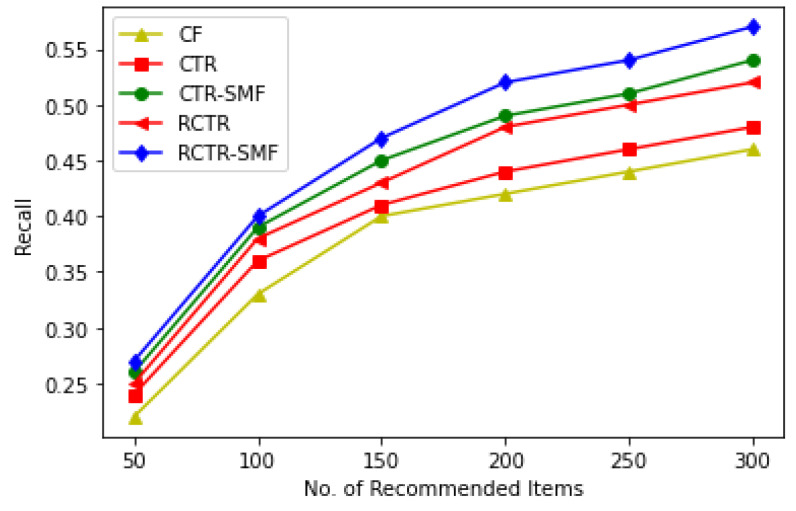
Overall Performance Comparison in terms of Recall and Number of Recommendations.

The overall performance for the in-matrix prediction task is presented in Table 3 and Figure 4, respectively. Here, the number of recommendations varies, i.e., *M* = {50, 100, 150, 200, 250, 300} and $\lambda_{v}$ is kept constant, i.e., $\lambda_{v}$ = 100. When the number of recommendations increases, the performance of RCTR–SMF improves. Figure 4 demonstrates that the proposed model outperforms all other methods with varied *M*. Moreover, the recall was observed to be too small across all the models on smaller values of *M*, especially when *M* < 50. The main reason is that in the testing data, the average number of artists per user is very low. That is why here in the present study *M* is kept to be not less than 50.

### 5.2. Influence of the Parameters $\lambda_{v}$, $\lambda_{q}$ and $\lambda_{r}$

This subsection attempts to discover the influence of various parameters, such as $\lambda_{v}$ (item content parameter), $\lambda_{q}$ (social relationship parameter) and $\lambda_{r}$ (item relational parameter) on the overall prediction performance of the proposed recommender system. The major focus is on balancing these parameters so that quality recommendations can be made to the target user.

**Figure 5:**
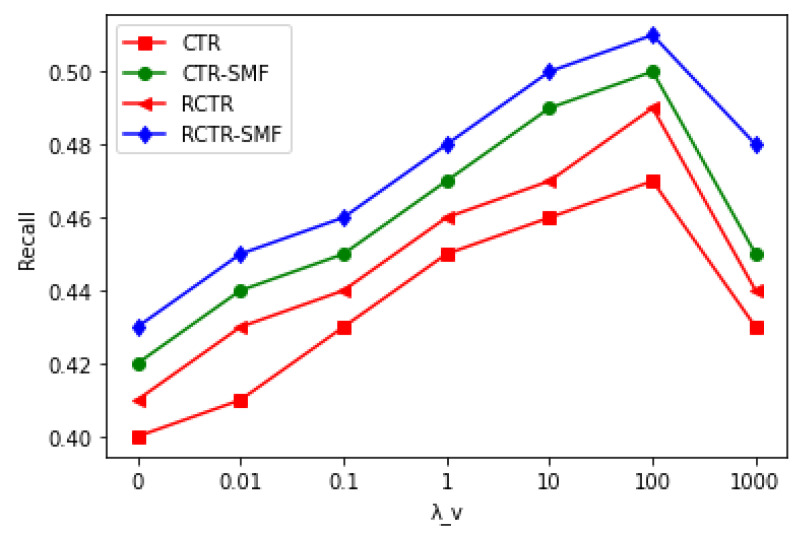
Recall comparison of CTR, CTR–SMF, RCTR and RCTR–SMF by varying $\lambda_{v}$ while keeping $\lambda_{q}$ and $\lambda_{r}$ constant @ M = 300.

First, the impact of $\lambda_{v}$ (precision parameter) was analyzed on all models in comparison, as shown in Table 4 and Figure 5, respectively. If $\lambda_{v}$ is small, then $v_{j}$ (item latent vector) diverges substantially from $\theta_{j}$ (topic proportions). The same effect was observed in all models. Figure 5 shows that RCTR–SMF outperforms all other models due to social network and item network information, as it can better represent the user-item latent space. Hence, the better the modelling of user likings and item tastes in latent space, the better the prediction performance.

If the social relationship parameter, i.e., $\lambda_{q}$ = 0, then the proposed model behaves more like RCTR, which heavily relies on item network structure (represented with link probability functions), item content information (represented with topic modelling) and the user-item interactions (represented with MF) to produce recommendations. If $\lambda_{q}$ = ∞, then the proposed model makes use of users’ social information only for modelling users’ preferences for prediction purposes.

On the other hand, if $\lambda_{r}$ = 0, then the proposed model collapses to CTR–SMF, which uses social information, item content, and user-item interactions. If $\lambda_{r}$ = ∞, then the proposed model makes use of item network information only for prediction purposes. It was observed that when $\lambda_{r}$ = 1, the RCTR–SMF model gives the best performance in terms of prediction accuracy.

**Figure 6:**
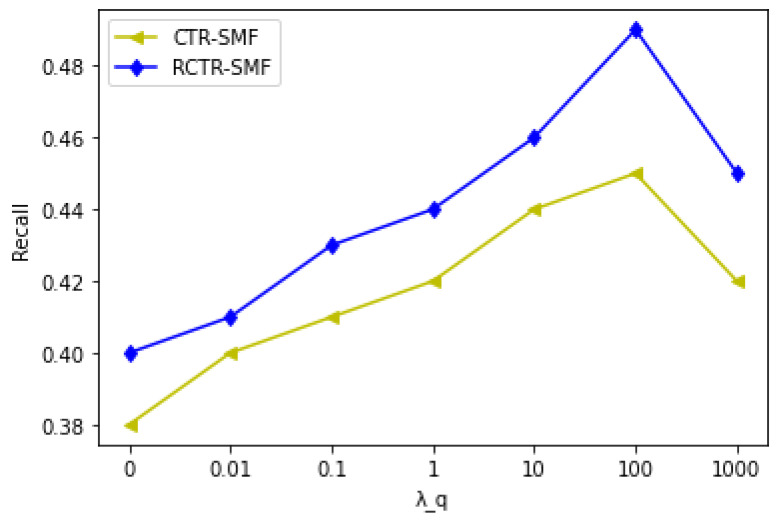
Recall Comparison of CTR–SMF and RCTR–SMF when $\lambda_{q}$ is varied, but $\lambda_{v}$ and $\lambda_{r}$ are kept constant @ M = 300.

Table 5 and Figure 6 exhibit the performance of the CTR–SMF and RCTR–SMF models for in-matrix predictions when $\lambda_{q}$ (social relationship parameter) is varied, but $\lambda_{v}$ (content parameter) and $\lambda_{r}$ (item relational parameter) are kept constant, respectively. From Figure 6, it can be concluded that $\lambda_{q}$ has a significant effect on the performance of the CTR–SMF and RCTR–SMF as the integration of social information with other information improves overall prediction accuracy. Figure 6 shows that RCTR–SMF consistently outperforms CTR–SMF. When $\lambda_{q}$ increases, the prediction accuracy of both models also increases. However, if $\lambda_{q}$ becomes larger beyond a certain threshold, the models rely more on social information than on other types of information and the overall system performance goes down. This means the prediction performance would not be reliable for larger values of $\lambda_{q}$.

**Figure 7:**
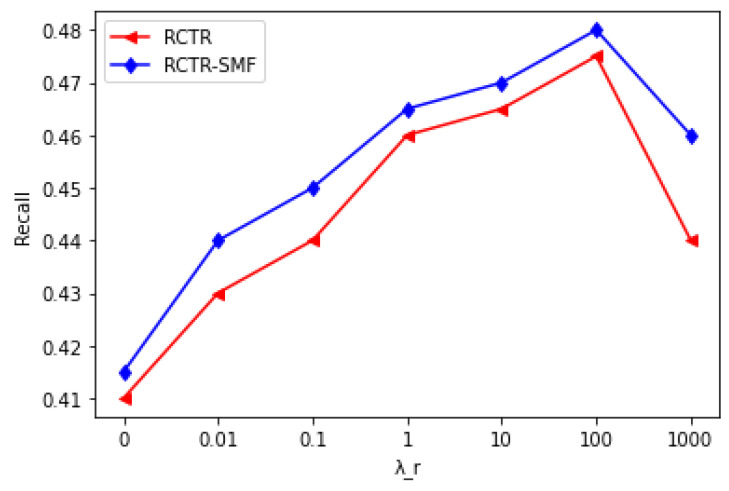
Recall Comparison of RCTR and RCTR–SMF when $\lambda_{r}$ is varied, but $\lambda_{v}$ and $\lambda_{q}$ are kept constant @ M = 300.

Table 6 and Figure 7 exhibit the performance of the RCTR and RCTR–SMF models for in-matrix predictions when $\lambda_{r}$ (item relational parameter) is varied, but $\lambda_{v}$ (content parameter) and $\lambda_{q}$ (social relationship parameter) are kept constant, respectively. Figure 7 demonstrates that $\lambda_{r}$ has a substantial influence on the performance of the RCTR and RCTR–SMF, as the integration of item network information with other information improves overall prediction accuracy. Figure 7 shows that RCTR–SMF consistently outperforms RCTR. When $\lambda_{r}$ increases, the prediction accuracy of both models also increases. However, if $\lambda_{r}$ becomes larger beyond a certain threshold, the models rely more on item relations information than on other types of information and the overall system performance goes down. This means the prediction performance would not be reliable for larger values of $\lambda_{r}$.

## 6. Conclusions

The work presented proposes a general hybrid hierarchical Bayesian model, known as the RCTR–SMF model, for recommender systems. This model recommends items to target users by exploiting a blend of different types of additional domain information such as social networking and item network structure in addition to item content and user-item interactions. RCTR–SMF also demonstrates the effectiveness of item relational network and social network information together in enhancing prediction accuracy. RCTR–SMF addresses the sparsity problem by utilizing the additional domain knowledge and alleviates the cold-start problem by making out-of-matrix predictions. RCTR–SMF can produce recommendations for new/inactive users and new/unseen items. Experimental evaluations on the RCTR–SMF model using a large real-world social media dataset confirm its effectiveness and outperformance in comparison with other existing state-of-the-art models. Based on the experiments, it can be determined that strong accuracy can be achieved by RCTR–SMF with a lesser number of learning iterations as compared to CTR but on par with CTR–SMF and RCTR.

For future work, RCTR–SMF can also deliver interpretable results based on the latent vectors of users that can prove to be useful for recommendation processes. Considering the scalability issue owing to the presence of millions of users and items in systems, a distributed learning algorithm can be developed for the proposed model that can seamlessly tackle the scalability issue of large-scale datasets. Traditional networks must be innovated to keep up with the swift change in the traffic flow of networks. Although there has been much advancement in storage devices, applications, and other computing resources, networks have for the most part remained unchanged. Software Defined Networking (SDN) is an approach that facilitates network management and configuration.

## Figures and Tables

**Table 1:** Description of the Lastfm Dataset.

Attributes	Total Count	Average(s) with Description
# Users	1892	
# Items	17,632	
# Tags	11,946	
# User-user relations	25,434	Average: 13,443 Social relations/user
# User—tags—items	186,479	Average: 98,562 tags/user
		Average: 14,891 tags/artist
		Average: 8764 different tags used for each artist
		Each user used an average of 18,930 different tags
# User-item relations	92,834	Each user listened to an average of 49,067 artists the most
		Each artist was listened to by an average of 5265 users

**Table 2:** List of Top 10 Recommendations.

Row	User ID	Artist ID	Artist Name	Prediction
0	45	1376	White Lies	0.884021
1	45	3739	Hole	0.860969
2	45	183	Jamiroquai	0.859264
3	45	1131	Tool	0.850992
4	45	3110	Fiona Apple	0.837827
5	45	3767	The Horrors	0.832549
6	45	428	The Libertines	0.832283
7	45	432	Klaxons	0.828805
8	45	1639	Jimi Hendrix	0.825787
9	45	324	Cobra Starship	0.813631

**Table 3:** Overall Performance Comparison.

Number of Recommendations	CF	CTR	CTR–SMF	RCTR	RCTR–SMF
50	0.22	0.24	0.26	0.25	0.27
100	0.33	0.36	0.39	0.38	0.4
150	0.40	0.41	0.45	0.43	0.47
200	0.42	0.44	0.49	0.48	0.52
250	0.44	0.46	0.51	0.5	0.54
300	0.46	0.48	0.54	0.52	0.57

**Table 4:** Recall comparison of CTR, CTR–SMF, RCTR and RCTR–SMF.

$\lambda_{v}$	CTR	CTR–SMF	RCTR	RCTR–SMF
0	0.4	0.42	0.41	0.43
0.01	0.41	0.44	0.43	0.45
0.1	0.43	0.45	0.44	0.46
1	0.45	0.47	0.46	0.48
10	0.46	0.49	0.47	0.5
100	0.47	0.50	0.49	0.51
1000	0.43	0.45	0.44	0.48

**Table 5:** Recall Comparison of CTR–SMF and RCTR–SMF.

$\lambda_{q}$	CTR–SMF	RCTR–SMF
0	0.38	0.40
0.01	0.40	0.41
0.1	0.41	0.43
1	0.42	0.44
10	0.44	0.46
100	0.45	0.49
1000	0.42	0.45

**Table 6:** Recall Comparison of RCTR and RCTR–SMF.

$\lambda_{r}$	RCTR	RCTR–SMF
0	0.41	0.415
0.01	0.43	0.44
0.1	0.44	0.45
1	0.46	0.465
10	0.465	0.47
100	0.475	0.48
1000	0.44	0.46

## Data Availability

There are no available data to be stated.
